# The role of ulnar nerve subcutaneous anterior transposition during open reduction and internal fixation of distal humerus fractures: a retrospective cohort study

**DOI:** 10.1007/s00264-020-04745-0

**Published:** 2020-10-03

**Authors:** Abdulaziz F. Ahmed, Ashik Mohsin Parambathkandi, Wai Jing Geraldine Kong, Motasem Salameh, Aiman Mudawi, Maamoun Abousamhadaneh, Yousef Abuodeh, Ghalib O. Ahmed

**Affiliations:** Orthopaedic Surgery Department, Hamad General Hospital, PO Box 3050, Doha, Qatar

**Keywords:** Distal humerus, Fracture, Ulnar nerve, Transposition, Neuropathy

## Abstract

**Purpose:**

To compare the rates of ulnar nerve neuropathy following ulnar nerve subcutaneous anterior transposition versus no transposition during open reduction and internal fixation (ORIF) of distal humerus fractures.

**Methods:**

This was a retrospective cohort study at an academic level I trauma centre. A total of 97 consecutive patients with distal humerus fractures underwent ORIF between 2011 and 2018. All included patients were treated with plates (isolated lateral plates excluded) and had no pre-operative ulnar neuropathy. Subcutaneous ulnar nerve anterior transposition was compared versus no transposition at the time of ORIF. The main outcome measure was the rate of ulnar nerve neuropathy. The secondary outcomes were the severity of the ulnar nerve neuropathy and the rate of ulnar nerve recovery.

**Results:**

Twenty-eight patients underwent subcutaneous ulnar nerve anterior transposition during ORIF, whereas 69 patients had no transposition. Transposition was associated with significantly higher rates of ulnar nerve neuropathy (10/28 versus 10/69; *P* = 0.027). An adjusted logistic regression model demonstrated an odds ratio of 4.8 (1.3, 17.5; 95% CI) when transposition was performed. Ulnar nerve neuropathy was classified as McGowan grades 1 and 2 in all neuropathy cases in both groups (*P* = 0.66). Three out of ten cases recovered in the transposition group, and five out of ten cases recovered in the no transposition group over a mean follow-up of 11.2 months (*P* = 1.00).

**Conclusion:**

We do not recommend performing routine subcutaneous ulnar nerve anterior transposition during ORIF of distal humerus fracture as it was associated with a significant 5-fold increase in ulnar nerve neuropathy.

## Introduction

The treatment of distal humerus fracture is often challenging due to their complexity and proximity to neurovascular structures [1]. At the time of open reduction and internal fixation (ORIF), it is paramount to mobilize and protect the ulnar nerve throughout the procedure to avoid iatrogenic injury [2]. Nonetheless, ulnar nerve neuropathy is a common complication after ORIF of distal humerus fractures, with a mean incidence of 12.3% [3].

There is sufficient evidence supporting subcutaneous anterior transposition of the ulnar nerve in patients with pre-operative signs of ulnar neuropathy [4, 5]. However, in patients with no pre-operative neurological deficit, the optimal handling of the ulnar nerve at the time of ORIF of the distal humerus remains controversial. Ruan et al. [5] found that ulnar transposition at the time of ORIF led to substantial improvement in cases that develop ulnar nerve neuropathy. On the other hand, other studies reported no added benefit [6, 7]or even an increased risk of ulnar nerve dysfunction [8].

This study aimed to compare the incidence of ulnar nerve neuropathy with or without subcutaneous anterior transposition of the ulnar nerve after ORIF of distal humerus fractures. The null hypothesis was there is no difference between ulnar nerve transposition and no transposition in the treatment of distal humerus fractures.

## Methods and materials

This study was reported by using the Strengthening the Reporting of Observational Studies in Epidemiology (STROBE) checklist for cohort studies [9].

### Study design

This study was approved by the institutional review board (MRC-01-19-101), and it was conducted at an academic level I trauma center. It was designed as a retrospective cohort comparing subcutaneous ulnar nerve anterior transposition with no transposition at the time of distal humerus fracture ORIF. The primary outcome was the rate of ulnar nerve neuropathy which was defined as any clinical findings of ulnar nerve transient or permanent sensory or motor signs or symptoms. The secondary outcomes were the severity of ulnar nerve neuropathy and the rate of ulnar nerve recovery. The severity of the ulnar nerve dysfunction was graded according to clinical findings of severity proposed by the McGowan classification [10].

### Eligibility criteria

**Fig. 1:**
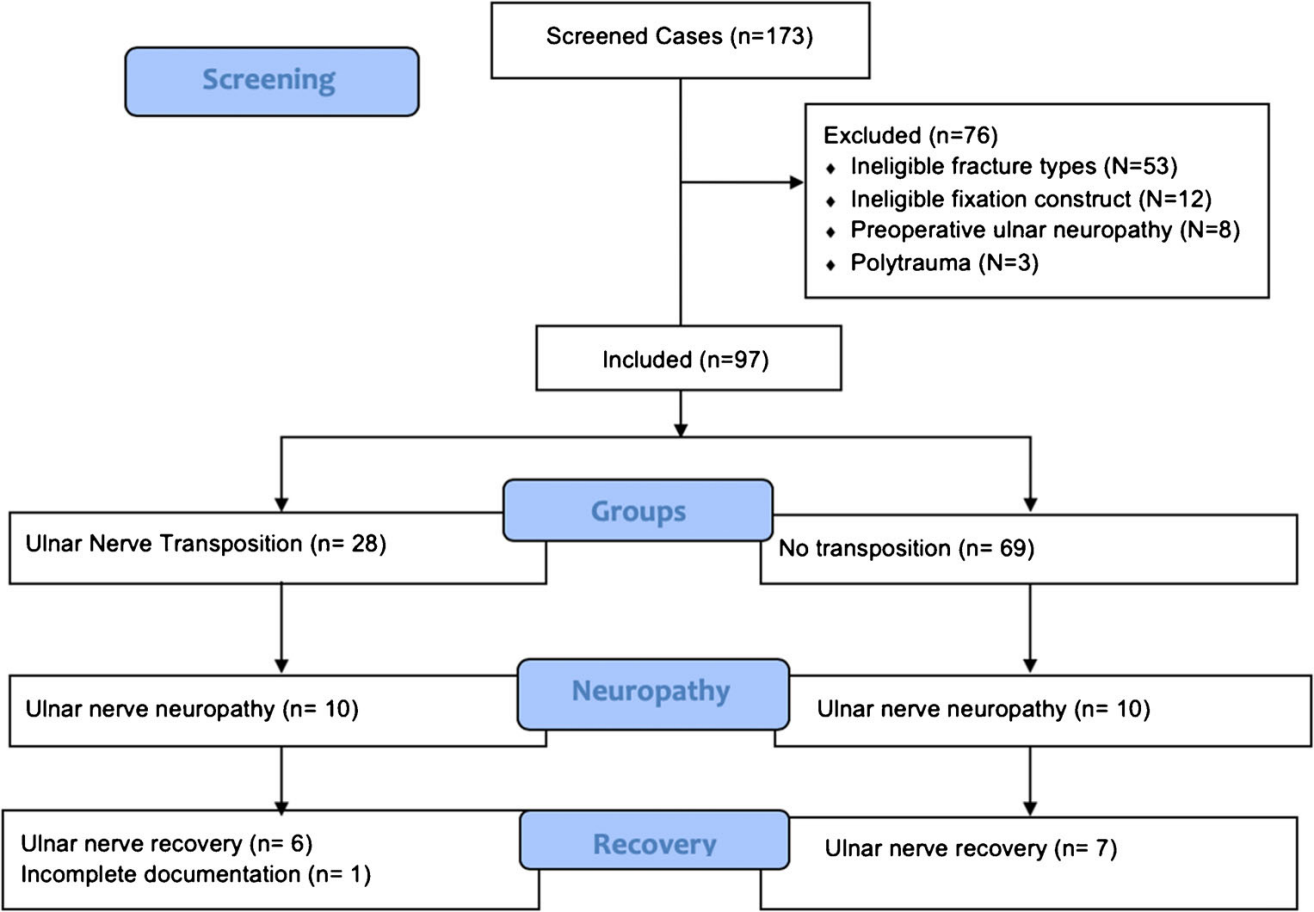
The study flowchart summarizing screening, inclusion, and follow-up of patients

The inclusion criteria were adults with distal humerus fracture cases that underwent ORIF with plates. The included fracture patterns were OTA/AO type 13A2, 13A3, 13B2, 13C1, 13C2, and 13C3. Fracture types 13A1, 13B1, and 13B3 were not included as medial plate fixation or ulnar nerve transposition is not warranted. The exclusion criteria were cases with pre-operative ulnar nerve neuropathy, previous central or peripheral neuropathy, treatment with a lateral plate only, treatment with a construct other than plates, and with incomplete documentation of pre-operative ulnar neuropathy (Fig. 1).

### Surgical techniques

**Fig. 2:**
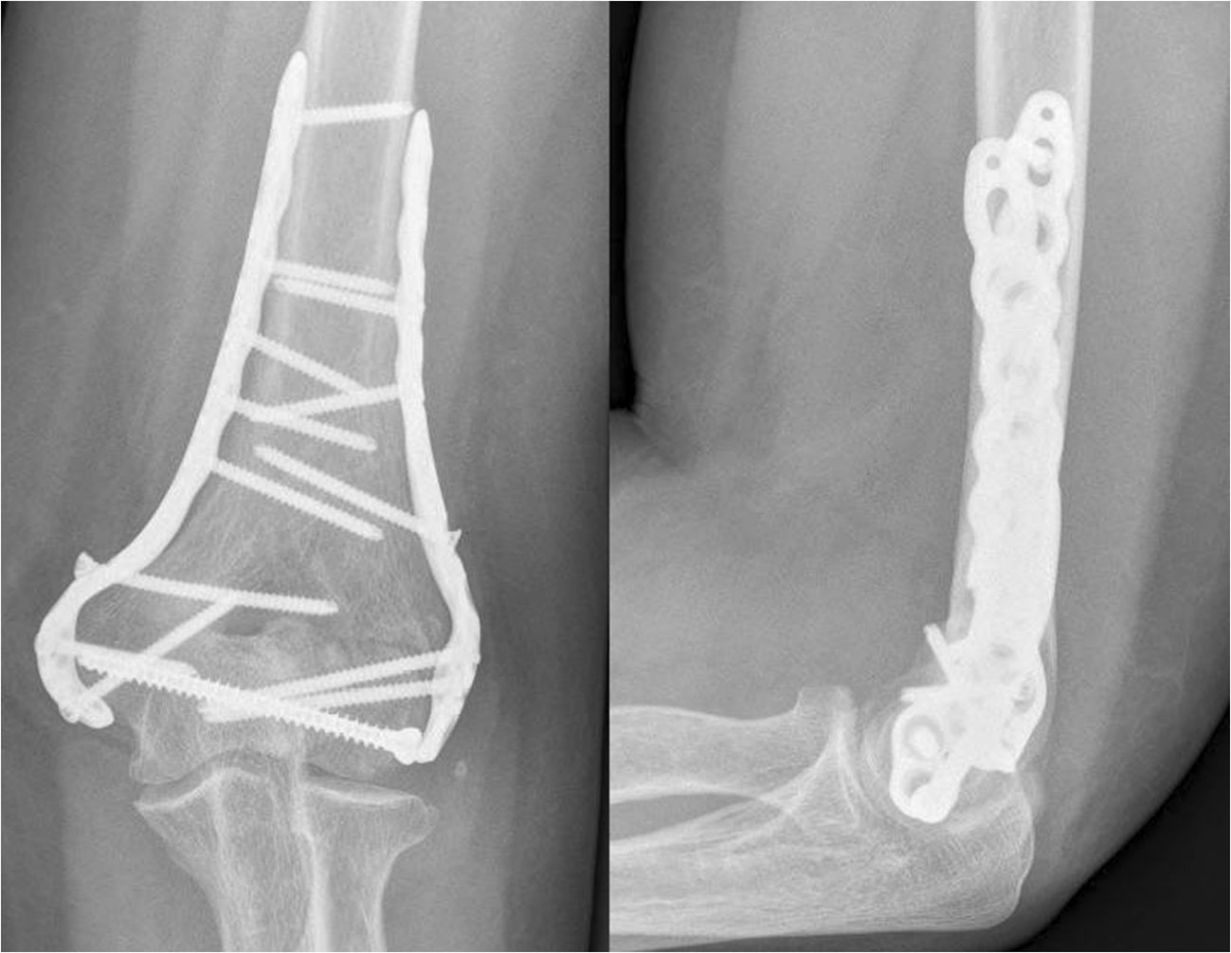
Anteroposterior and lateral elbow radiographic views of 180° parallel plating of a distal humerus fracture

**Fig. 3:**
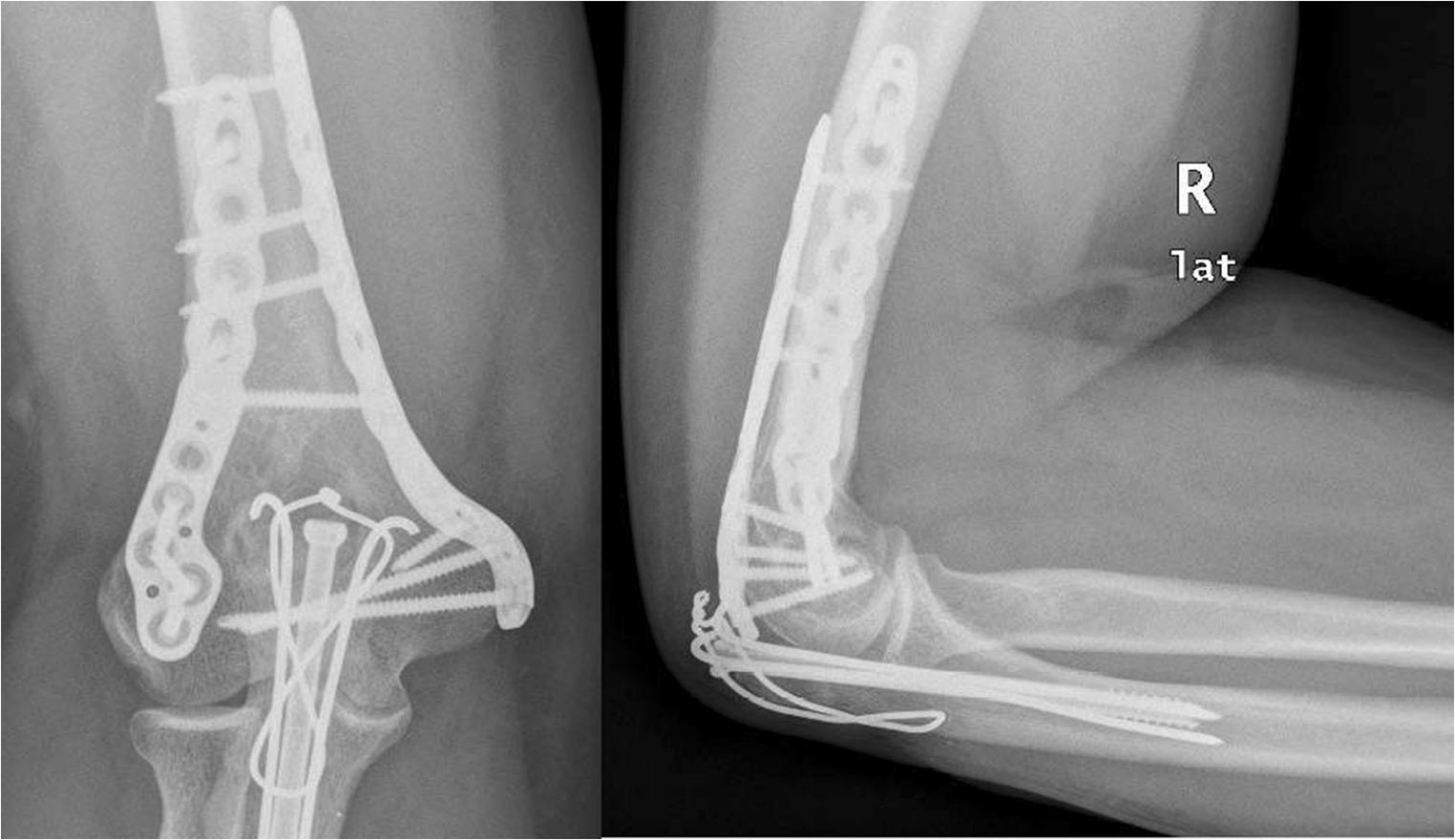
Anteroposterior and lateral elbow radiographic views of 90°–90° orthogonal plating of a distal humerus fracture

A posterior approach to the elbow was utilized in most fracture patterns. A triceps-sparing approach with medial and/or lateral windows or an olecranon osteotomy was used depending on the surgeon’s discretion and the fracture comminution for OTA/AO type 13A or 13C fractures. However, a medial elbow approach was used for OTA/AO 13B fractures which involved isolated medial columns. Fixation was achieved with several plating configurations which were both dependent on the surgeon’s intra-operative decision and the fracture configuration. Isolated medial plating was used in specific OTA/AO type 13A or 13B medial column isolated fractures. Parallel 180° (Fig. 2) or orthogonal 90°–90° (Fig. 3) plating techniques were reserved for the vast majority of OTA/AO type 13A and 13C fractures.

**Fig. 4:**
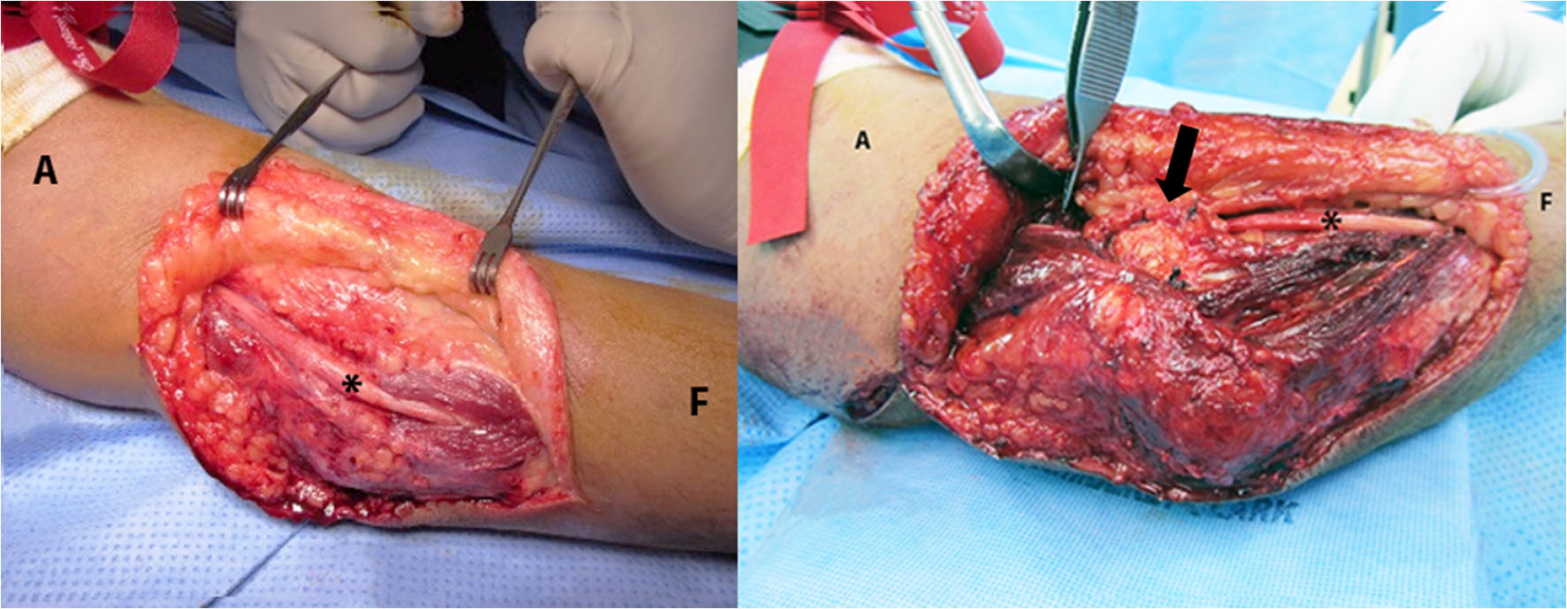
An intraoperative image demonstrating (right) ulnar nerve in situ decompression and (left) anterior subcutaneous ulnar nerve transposition. A, arm; F, forearm; (*), ulnar nerve; black arrow, transposed ulnar nerve with fascial wrapping

In all procedures, the ulnar nerve firstly decompressed at the cubital tunnel, followed by careful dissection proximally along the medial border of the triceps. The ulnar nerve was mobilized throughout the procedure with a vessel loop or a Penrose drain. At the conclusion of the surgery, the ulnar nerve is either managed with subcutaneous anterior transposition or with no transposition and retaining the nerve in its original position (Fig. 4). In the non-transposition group, the ulnar nerve was returned to its original position behind the medial epicondyle, whereas the subcutaneous anterior transposition group entailed the utilization of fascial wrapping. Firstly, the ulnar nerve was ensured to be easily transposable anteriorly. The fascia of the flexor pronator mass origin was exposed and separated of the overlying subcutaneous tissues. Approximately a 2 × 2-cm trap door of fascia was created with its base attached to the medial epicondyle. Afterward, the ulnar nerve was transposed anteriorly, and the fascial flap was sutured to the overlying subcutaneous tissues, thus covering the ulnar nerve. The surgeons who performed the procedures were attending trauma orthopedic surgeons. The decision for transposition or type of plating was at the surgeon’s discretion.

### Data source and collection

The data source was our institution’s electronic medical records. The operative database was searched from 2011 until 2018 with the keywords “humerus” and “fractures” to identify all potential cases. The pre-operative assessments, operative notes, and post-operative assessments were reviewed.

The baseline variables that were collected included age, sex, fracture type according to the OTA/AO classification, open/closed fractures, ipsilateral upper limb injuries, presence of polytrauma, number of procedures, plate configuration, and the use of an olecranon osteotomy.

Exposure variables were either subcutaneous anterior transposition of the ulnar nerve or no transposition. Outcome variables such as the rate of post-operative ulnar nerve neuropathy were documented post-operatively through physical examination. The ulnar nerve neuropathy was graded according to the McGowan classification [10] into three grades: grade 1 indicating subjective ulnar nerve sensory symptoms; grade 2 as objective ulnar nerve sensory symptoms and weakness with or without hand intrinsic muscle atrophy; and grade 3 as severe sensorimotor deficit with paralysis of the hand muscle supplied by the ulnar nerve. Other outcome variables included the rate of ulnar neuropathy recovery and reoperations. Due to the retrospective nature of this study, we could not obtain electromyographic evidence of post-operative neuropathic dysfunction.

### Statistical analysis

The statistical analysis was performed with Stata/IC (StataCorp. 2019. Stata Statistical Software: Release 16. College Station, TX: StataCorp LLC.). Continuous variables were reports with means and SD, and dichotomous variables were reported as proportions. The two group *t* test or the Mann-Whitney *U* test was used for comparing continuous variables, whereas the chi-square or the Fischer’s exact tests were used for comparing proportions depending on the normality of the data. A logistic regression model was designed to evaluate the effect of ulnar nerve transposition on the development of ulnar nerve dysfunction while adjusting for age, sex, fracture type, presence of open fractures, polytrauma, ipsilateral limb injuries, and plate configuration. The effect of ulnar nerve transposition or not was estimated with odds ratio (OR) at a 95% confidence interval (CI). In addition, a difference with a *P* value < 0.05 was considered statistically significant.

## Results

### Participants

A total of 173 cases were identified in our database search that was labeled as distal humerus fractures. Of the total identified patients, 76 patients were excluded as they did not meet the eligibility criteria. The reasons for exclusion were ineligible fracture types in 53 patients, treated with a construct other than dual plates or isolated medial plates in 12 patients, pre-operative ulnar nerve neuropathy in eight patients, and three patients were polytraumatized and treated while intubated. Thus, the total number of eligible patients for inclusion was 97 cases with distal humerus fractures (Fig. 1). Of the total included sample, 28 patients underwent subcutaneous ulnar nerve anterior transposition at the time of ORIF, whereas 69 patients had no nerve transposition.

### Descriptive data

Patients’ characterstics are summarized in Table 1. The mean age was 35.8 years (17–82) with most patients being males (75%). Most fractures were closed (69%) with the most prevalent fracture type being OTA/AO type C1 followed by C2 and C3. Fracture types OTA/AO A2, A3, and B2 were less common. Regarding associated injuries, polytrauma was present in 28%, and associated ipsilateral upper limb injuries were present in 20%.

**Table 1:** Baseline patients’ characteristics

	Transposition (*N* = 28)	No transposition (*N* = 69)	*P* value
Age	35 ± 11	36 ± 14.2	0.7^†^
Sex			0.6
Males	20 (71.4%)	53 (76.8%)	
Females	8 (28.5%)	16 (23.2%)	
Fracture type			0.8
13A2	1 (3.6%)	5 (7.3%)	
13A3	1 (3.6%)	2 (2.9%)	
13B2	4 (14.3%)	5 (7.2%)	
13C1	10 (35.7%)	24 (34.8%)	
13C2	6 (21.4%)	21 (30.4%)	
13C3	6 (21.4%)	12 (17.4%)	
Open fractures	6 (21.4%)	22 (31.8%)	0.34
Ipsilateral limb injuries			0.38
Clavicle fracture	1 (3.6%)	0	
Proximal humerus fracture	0	1	
Proximal radius and ulna fractures	1	1	
Forearm shaft fractures	0	4	
Distal radius fractures	2	6	
Polytrauma	8 (28.6)	20 (28.9)	1
Plate configuration			**0.02**
Orthogonal 90°–90°	7 (25%)	36 (52.2%)	
Parallel 180°	17 (60.7%)	30 (43.5%)	
Medial plate only	4 (14.3%)	3 (4.35%)	
Olecranon osteotomy			
Yes	16 (57.1%)	27 (39.1%)	
No	10 (35.7%)	41 (59.4%)	0.09
Olecranon fracture	1 (3.6%)	1 (1.45%)	

All statistical comparisons were performed with a Fischer’s exact test unless stated otherwise

Bold text signifies a significant *P* value

^†^Two group *t* test

Orthogonal 90°–90° plating was the most common plate configuration in the no transposition group (52.2% versus 25%); however, parallel plating 180° was significantly more prevalent in the transposition group (60.7% versus 43.5%). Isolated medial plating was the least utilized plating configuration. An olecranon osteotomy was utilized in 44.3% of cases, 52.5% had no olecranon osteotomy, and 2% of fractures had an associated olecranon fracture.

### Outcome data

Out of the 97 patients, 20 (20.6%) patients had documented post-operative ulnar nerve neuropathy, whereas the rest of 77 patients had clear documentation of intact post-operative neurological function. The transposition group had a significantly higher ulnar nerve neuropathy (*P* = 0.027; Fischer’s exact test) when compared with no transposition (10 out of 28 versus 10 out of 69) (Table 2). Patients who underwent ulnar nerve transposition had an OR of 3.3 (1.02, 10.2; 95% CI) for developing ulnar nerve neuropathy. A logistic regression model adjusted for age, sex, fracture type, open fractures, polytrauma, the use of an olecranon osteotomy, and plate configuration demonstrated a further increase in ulnar nerve dysfunction following transposition (Table 3). The adjusted OR for ulnar nerve neuropathy in patients who underwent transposition was 4.8 with a significant 95% CI (1.3, 17.5) while holding other variables constant. The added covariates were not predictive of ulnar nerve neuropathy as per the adjusted logistic regression model.

**Table 2:** Comparison of ulnar nerve transposition versus no transposition for the rate of ulnar nerve neuropathy, the severity of neuropathy, ulnar nerve recovery, and follow-up

Outcome	Transposition (*N* = 28)	No transposition (*N* = 69)	*P* value
Ulnar nerve neuropathy	10 (35.7%)	10 (14.5%)	**0.027**^†^
McGowan grade			0.66^‡^
Grade 1	5	6	
Grade 2	5	4	
Grade 3	0	0	
Ulnar nerve recovery			1.00^†^
Recovered	6	7	
Persistent	3	3	
Not documented	1	0	
Mean follow-up	13.2 months	10.7 months	0.9^‡^

A bold text signifies a statistical significant value

^†^Fischer’s exact test; ^‡^Mann-Whitney *U* test

**Table 3:** Adjusted analysis performed with a multivariate logistic regression model for ulnar nerve neuropathy

Factor	Odds ratio	*P* value	95% CI
Age	1.02	0.2	0.98, 1.07
Sex			
Male	2.1	0.26	0.57, 7.7
Female	-	-	-
Fracture type			
13A	-	-	-
13B	0.57	0.75	0.02, 17.7
13C	2.4	0.5	0.18, 30.5
Open fracture			
Yes	1.01	0.98	0.27, 3.8
No	-	-	-
Polytrauma			
Yes	0.77	0.72	0.19, 3.05
No	-	-	-
Plate configuration			
Orthogonal 90°–90°	0.9	0.95	0.04, 17.6
Parallel 180°	0.3	0.44	0.013, 6.34
Medial plate	-	-	-
Olecranon osteotomy			
Yes	1.48	0.54	0.41, 5.36
No	-	-	-
Ulnar nerve transposition			
Yes	4.8	**0.017**	**1.3, 17.5**
No	-	**-**	**-**

Included covariates: age, sex, OTA/AO fracture type, open or closed fracture, presence of polytrauma, plate configuration, the use of an olecranon osteotomy, and ulnar nerve transposition. *CI* confidence interval

Bold text signifies a statistical significant value

Eleven and nine cases were classified as McGowan grades I and II, respectively, without significant difference between the transposition and no transposition groups (*P* = 0.66; Mann-Whitney *U* test). The ulnar nerve neuropathy resolved in 12 (65%) cases out of 20 at a mean follow-up period of 11.2 months. One case was excluded from the follow-up due to incomplete documentation. Six out of 10 cases recovered in the transposition group, and seven out of 10 cases recovered in the group without transposition (*P* = 1.00; Fischer’s exact test).

## Discussion

In this retrospective study, the overall rate of post-operative ulnar nerve neuropathy was 20.6%, with the subgroup rates being 35.7% and 14.5% for transposition and no transposition, respectively. More notably, performing an ulnar nerve transposition at the time of ORIF was associated with a significant five fold increase in the risk of developing ulnar nerve neuropathy when adjusting for several factors including age, sex, fracture type, open fractures, polytrauma, the use of an olecranon osteotomy, and plate configuration. The recovery of ulnar nerve dysfunction was noted in 65% of patients (13 out of 20) with no difference between both groups.

The treatment of distal humerus fractures is technically demanding due to the proximity of neurovascular structures and due to the complexity of the fracture patterns. In a recent meta-analysis, the incidence of ulnar nerve neuropathy has been reported as 19% following ORIF for distal humerus fractures which are comparable with the incidence reported in this study [11]. The ulnar nerve dysfunction can occur pre-operatively, at the time of surgery or post-operatively. Intra-operative factors include iatrogenic injury related to the ulnar nerve handling or direct injury, hardware irritation, or scar tissue formation around the ulnar nerve. In our study, the most plausible cause of dysfunction attributed to nerve handling and transposition, as we have excluded pre-operative neuropathy.

The operative management of the ulnar nerve without pre-operative dysfunction during ORIF can be either treated with subcutaneous anterior transposition or by retaining the nerve in its original position, however, which method is effective remains a matter of debate. Ruan et al. reported that 29 out of 117 patients with distal humerus fractures had ulnar nerve dysfunction pre-operatively [5]. Of the 29 patients, ulnar transposition at the time of ORIF resulted in 86.7% good to excellent improvement when compared with 57% in the in situ decompression group. Likewise, McKee et al. performed ulnar nerve transposition in 21 elbows with ulnar nerve dysfunction following elbow surgical fixation, where good-to-excellent outcomes were achieved in 17 out of the 21 patients with ulnar nerve transposition [4]. Therefore, routine ulnar nerve transposition was thought to be a preventative measure for the development of ulnar nerve neuropathy following distal humerus fractures. However, the role in fractures without pre-operative nerve dysfunction is controversial, with the theoretical advantage of less ulnar nerve injury extrapolated from these aforementioned studies.

Despite this prophylactic notion for transposition, several studies have found that transposition did not reduce the rate of post-operative ulnar nerve neuropathy. In a study on 107 patients with distal humerus fractures, Wiggers et al. found that 17% of cases had neuropathy regardless of whether the nerve was transposed or not (*P* = 0.43) [7]. Additionally, Worden et al. and Vazquez et al. reported no benefit of ulnar nerve transposition over in situ decompression groups in reducing the rates of ulnar nerve dysfunction [6, 12]. In contrast, some studies have found that ulnar transposition during distal humerus ORIF increases the risk of ulnar nerve neuropathy. Chen et al. investigated the fate of ulnar nerve at the time of ORIF of distal humerus fractures in 137 patients and reported that transposition had four times the incidence of ulnar nerve neuropathy when compared with no transposition (*P* < 0.001) [8]. Likewise, we found that ulnar nerve transposition had 4.8 times the risk for developing post-operative dysfunction, and therefore, we do not recommend performing nerve transposition in cases without pre-operative neuropathy.

Ulnar nerve neuropathy severity following ORIF of distal humerus fracture is variable and reported according to the McGowan classification in three studies. Wiggers et al. [7] reported only McGowan grade 3 neuropathy as per their reporting criteria, whereas the other studies reported McGowan grade 1 (56–72%) as the most frequent neuropathy followed by grade 2 (28–44%), with no grade 3 cases [6, 12]. In this study, McGowan grade 1 and grade 2 represented 55% and 45% of post-operative ulnar nerve neuropathy, respectively. No cases with McGowan grade 3 neuropathies were reported.

There are several limitations to this study that should be acknowledged due to the retrospective design. Selection bias might have been introduced due to the non-systematic decision by the operating surgeon to perform transposition. The data collection process was dependent on the accuracy of follow-up progress in-hospital and office notes without evidence of neuropathy on electromyographic studies, which can lead to non-differential information bias such as misclassifying ulnar nerve dysfunction and its severity. In addition, we could not collect the exact time point when an improvement in ulnar nerve symptoms was noted. Another limitation is that it is almost impossible to measure how delicately the nerve was handled during surgery, and this undoubtedly has an effect on the rate of ulnar nerve dysfunction. Furthermore, a post-hoc power analysis demonstrated a study power of 48% to detect a significant difference between both groups for the development of ulnar nerve neuropathy, which might increase the risk of type II error. However, the paucity of distal humerus fractures precluded the ability to increase the sample size to achieve sufficient study power. Finally, we had a short follow-up of 11 months; however, we assumed this follow-up period is sufficient to detect the occurrence for most neuropathies and the change in their post-operative course.

In conclusion, transposition of the ulnar nerve during ORIF of distal humerus fractures without pre-existing neuropathy is associated with almost five times the risk for developing ulnar nerve neuropathy when compared with no transposition. The risk of ulnar nerve neuropathy was not significantly influenced by other confounding factors. Therefore, we do not recommend performing routine ulnar nerve transposition during ORIF of distal humerus fractures. The benefit of ulnar nerve transposition with or without pre-existing ulnar neuropathy in distal humerus fractures still needs to be elucidated in prospective and preferentially randomized studies.
